# Long-Term Effects of Exposure to Ionizing Irradiation on Periodontal Health Status – The *Tinea capitis* Cohort Study

**DOI:** 10.3389/fpubh.2015.00226

**Published:** 2015-10-19

**Authors:** Siegal Sadetzki, Angela Chetrit, Harold D. Sgan-Cohen, Jonathan Mann, Tova Amitai, Hadas Even-Nir, Yuval Vered

**Affiliations:** ^1^Cancer and Radiation Epidemiology Unit, Gertner Institute for Epidemiology and Health Policy Research, Chaim Sheba Medical Center, Tel Hashomer, Israel; ^2^Sackler Faculty of Medicine, Tel Aviv University, Tel Aviv, Israel; ^3^Department of Community Dentistry, Faculty of Dental Medicine, The Hebrew University Hadassah Medical School, Jerusalem, Israel

**Keywords:** public health, dental, ionizing radiation, periodontal disease, risk assessment, risk factor

## Abstract

Studies among long-term survivors of childhood cancer who had received high-dose irradiation therapy of 4–60 Gy, demonstrated acute and chronic dental effects, including periodontal diseases. However, the possible effects of low to moderate doses of radiation on dental health are sparse. The aim of this study is to investigate the association between childhood exposure to low–moderate doses of ionizing radiation and periodontal health following 50 years since exposure. The study population included 253 irradiated subjects (treated for *Tinea capitis* in the 1950s) and, 162 non-irradiated subjects. The estimated dose to the teeth was 0.2–0.4 Gy. Dental examination was performed according to the community periodontal index (CPI). Socioeconomic and health behavior variables were obtained through a personal questionnaire. Periodontal disease was operationally defined as “deep periodontal pockets.” A multivariate logistic regression model was used for the association of irradiation status and other independent variables with periodontal status. The results showed that among the irradiated subjects, 23%, (95% CI 18–28%) demonstrated complete edentulousness or insufficient teeth for CPI scoring as compared to 13% (95% CI 8–19%) among the non-irradiated subjects (*p* = 0.01). Periodontal disease was detected among 54% of the irradiated subjects as compared to 40% of the non-irradiated (*p* = 0.008). Controlling for education and smoking, the ORs for the association between radiation and periodontal disease were 1.61 (95% CI 1.01–2.57) and 1.95 (95% CI 1.1–3.5) for ever never and per 1 Gy absorbed in the salivary gland, respectively. In line with other studies, a protective effect for periodontal diseases among those with high education and an increased risk for ever smokers were observed. In conclusion, childhood exposure to low-moderate doses of ionizing radiation might be associated with later outcomes of dental health. The results add valuable data on the long-term health effects of exposure to ionizing radiation and support the implementation of the ALARA principle in childhood exposure to diagnostic procedure involving radiation.

## Introduction

Periodontal disease is an inflammatory pathology complex, combining distinct, pathological entities caused by the interaction of dental bacterial plaque and the host, affecting the soft and hard structures that support the teeth. Dental plaque, smoking and tobacco use, stress, genetic disorders, systemic infections, diseases, and other factors predisposing to plaque accumulation, are considered to be risk factors (1, 2). According to the World Health Organization (WHO), periodontal disease is considered to be one of the most common pathologies of humans (3, 4), and a recent systemic analysis of global burden of oral conditions has demonstrated an increased trend of severe periodontitis from 1990 to 2010 (5).

Exposure of the head and neck to therapeutic levels of ionizing radiation can cause a wide spectrum of acute and chronic oral complications. The degree of damage is dependent on factors related to the treatment regime, which includes irradiation field, fractionation, total dose absorbed, and the age of the patient at time of therapy (6). The most common acute side effects of irradiation to the head and neck area are vascular damage, salivary gland dysfunction, oral mucositis, infection, taste dysfunction, and pain. The most common chronic side effects are mucosal fibrosis and atrophy, xerostomia, dental caries, soft tissue necrosis, and osteonecrosis (6–8).

Studies among long-term survivors of childhood cancer who had received irradiation therapy of 4–60 Gy, demonstrated late dental and maxillofacial effects (9, 10). The dental abnormalities included foreshortening and blunting of roots, incomplete calcification, premature closure of dental apices, delayed or arrested tooth development, crown defects, irradiation caries, and one or both parotid glands showing absent secretions. Other studies among long-term survivors of childhood cancer who had received irradiation therapy of 18–24 Gy, demonstrated arrested root development (11), and high risk of developing periodontal disease (12). The defects were more severe in those patients who received irradiation at an earlier age and at higher dosages.

Between 1946 and 1960, about 20,000 Israeli children were treated with ionizing radiation to the head for *Tinea capitis* (TC), a benign fungal disease of the scalp (13, 14). Epilation by means of ionizing radiotherapy, in addition to manual plucking of the hair, was considered as the most efficacious treatment of TC at that period of time (15, 16). This population was composed mostly of new immigrants from North Africa and to a lesser extent from the Middle East. In 1968, a comprehensive follow-up of a cohort including the irradiated group and two comparison groups was initiated and delayed side effects of irradiation were reported (17, 18).

Although radiotherapy for treatment of TC has long been replaced with oral and topical medication, exposure to medical radiation remains a contemporary concern due to the frequent use of procedures, such as computed tomography (CT), especially in children. The present study population provided an opportunity to investigate the effects of early exposure to low to moderate doses of ionizing irradiation, on periodontal health, following a long period of 50 years. We hypothesize that the non-exposed will present better indicators for periodontal health status compared to the irradiated group.

## Materials and Methods

The TC cohort was comprised of 10,834 Jewish subjects irradiated for TC in Israel from 1948 to 1960. The comparison group included 10,834 non-irradiated subjects derived from the National Population Registry and individually matched to the exposed subjects by age (±2 years), gender, country of birth, and year of immigration to Israel. The TC Study cohort has been described and analyzed in several previous studies (14, 17–19). The analysis presented here is based on a random subsample of irradiated and non-irradiated subjects from the TC cohort. The inclusion criteria were residency in two large cities in Israel and being free of malignant disease. Exclusion criteria included death and medical conditions that did not allow an interview (e.g., mental disease, CVA, etc.). The sample of individuals who comply with these eligibility criteria included 827 individuals (426 irradiated and 401 non-exposed population controls).

Treatment of TC had involved application of the Adamson–Kienbock technique. Prior to irradiation, the subject’s hair was shaved and remaining hair was removed by a waxing process. The scalp area was then divided into five fields, each treated on one of five consecutive days. The majority of patients received one course of therapy (over 5 days). Approximately 9% of the patients underwent two or more courses.

In the 1960s, dosimetry was estimated retrospectively using one of the original X-ray machines and a specially designed head phantom. Doses absorbed by the brain were measured with the aid of ionization chambers. The results were presented as doses absorbed at several grid points inside the phantom head in two horizontal layers 2.5 cm apart (20). In the 1980s, doses to the brain for individual patients were calculated. These estimations were based on the measurements made on an anthropomorphic phantom, the prescribed medical center-specific exposure technique, the number of treatment courses and age and gender (which were highly correlated with size of the child).

The mean average dose for all irradiated individuals, to the brain, was found to be 1.5 Gy (range: 1.0–6.0 Gy), and to the thyroid gland 0.09 Gy (range: 0.04–0.5 Gy) (16, 17, 20). The dose to the parotid gland in our TC cohort was found to be 0.78 Gy (range of 0.63–2.9 Gy) (21), and the estimated dose to the teeth ranged between 0.2 and 0.4 Gy, with a higher dose to the posterior molars (Marilyn Stovall, personal communication).

The study protocol was approved by the ethics panel of the Chaim Sheba Medical Center and informed consent was obtained from all participants. Data were gathered during individual meetings that included an interview and a dental examination performed by one senior dental epidemiologist (Yuval Vered) who was blind to the irradiation status of the examinees. Most meetings were held at a dental clinic, while 82 subjects (who were unable to reach the clinics) were examined and interviewed at home. The questionnaire covered details about socio-demographic parameters, health behavior variables (smoking and alcohol use), history of diseases among the subjects and their family, past hospitalizations, use of medications and past exposure to irradiation (diagnostic and/or therapeutic and/or occupational). This questionnaire had been used in several previous TC studies (22). A dental questionnaire was added including questions regarding dental care services utilization, current oral hygiene behavior, and current self-perceived mouth dryness (self-perception, need to drink water at night, need to lubricate mouth, and suffering from dry mouth during speech).

The results of the clinical periodontal examinations were recorded employing the community periodontal index (CPI) with the specially designed probe (23). Three indicators of periodontal status were used for this assessment: gingival bleeding, calculus, and periodontal pockets. The index scale was nominal: 0 = healthy; 1 = bleeding; 2 = calculus; 3 = “shallow” periodontal pocket of 4–5 mm; 4 = “deep” periodontal pocket above 6 mm. The mouth was divided into six sextants (tooth numbers: 18–14, 13–23, 24–28, 38–34, 33–43, 44–48), and index teeth to be examined were 17, 16, 11, 26, 27, 37, 36, 31, 46, and 47. No radiographs were taken.

The worst CPI score was given to each sextant followed by the worst CPI score for each person. Percentages of these scores were then calculated for the entire group. The outcome-dependent variable was operationally defined as CPI score 4 (“deep” periodontal pocket), which denote severe chronic periodontal disease. This categorization is in accordance with that of the modified WHO guidelines for CPI and is commonly utilized as the preferred operative clinical definition for periodontal disease. In line with this definition, the outcome was treated as a dichotomous variable and the analysis was performed using simple logisti regression.

In addition, periodontal health status findings were presented and analyzed as the mean number of sextants (total of six) with each CPI score.

Demographic characteristics of the irradiated and non-irradiated participants were compared using the Chi Square test for discrete variables. *T*-test was used for the comparison between the mean age of the participants and non-participants. Wilcoxon non-parametric test was performed for the comparison of number of missing sextants between irradiated and non-irradiated.

The main independent variable, irradiation status, was categorized dichotomously (yes/no). Other independent variables included gender, age at interview, education (up to 9 years, high school, academic/college degree), income compared to the national average income (much less, less, similar, more and much more), and self-defined religiosity (secular, traditional, religious and orthodox). Smoking, history of diabetes, dental visit during the last year, and brushing teeth behavior were also investigated. Differences in the study parameters by periodontal disease were assessed using Chi Square test for discrete variables.

To evaluate the independent effect of each study variable on periodontal disease (“worst” CPI score 4 – deep periodontal pockets), a multivariate logistic regression analysis was performed. Predictors with a *p*-value of 0.2 or less in the univariate analysis were included in the multivariate regression. A *p*-value cut-off of 0.2 was used to detect variables that may not be significantly associated to the outcome in the univariate analysis and may be significant in a multiple model after adjustment of other covariates. Variables that did not reach statistical significance in the regression models were excluded using a backward elimination method. An additional logistic regression analysis was performed to assess the risk for periodontal disease per dose unit using the absorbed doses to the salivary gland. Linear dependency of the risk on dose was checked, introducing the term of dose^2^ in the linear quadratic model and testing its significance using the Wald test. All of the statistical analyses were done with Statistical Analysis System software version 9.1 (SAS Institute, Inc., Cary, NC, USA). Statistical significance was set at *p* < 0.05, using two-tailed tests.

## Results

Of the initial study sample of 827 subjects, 20% (*n* = 171) could not participate in the study due to unavailable addresses; of the remaining study population, 415 individuals were interviewed (79 and 48% of the irradiated and non-irradiated groups, respectively). Participants and non-participants of each study group did not differ significantly by gender and place of birth, and by age in the irradiated group. Participants of the non-irradiated group tended to be slightly younger than non-participants [mean ages 58 (SD 4.3) vs. 59 (SD 4.4) years, respectively; *p* = 0.02]. Fifty-nine subjects (14 irradiated cases and 45 controls) completed a refusal interview. No significant differences in smoking, education, self-reported caries, and periodontal status were observed between the controls who participated in the study and those who refused a full interview and dental examination, but participated in the refusal interview (*p* = 0.8, 0.9, 0.3, and 0.6, respectively). Although the number of cases who completed the refusal questionnaire was small, similar findings were observed.

Table 1 presents the distribution of the 253 irradiated and 162 non-irradiated participants by socio-demographic variables, smoking, prevalence of diabetes, and dental-related variables. The age of most study participants was 55–64 years. Significant statistical differences between the groups were detected for education and income, and for brushing teeth habits.

**Table 1:** **Distribution of independent variables among study population by irradiation status**.

	Irradiated subjects (*n* = 253)		Non-irradiated subjects (*n* = 162)		*p*
	*n*	%	*n*	%	
**Gender**
Male	118	46.6	68	42.0	0.4
Female	135	53.4	94	58.0	
**Age**
49–54	37	14.7	32	19.8	0.3
55–59	101	40.1	69	42.6	
60–64	87	34.5	50	30.9	
65–72	27	10.7	11	6.8	
**Family status**
Married	194	76.7	121	74.7	0.2
Single	5	2.0	1	0.6	
Separate/divorced	29	11.5	28	17.3	
Widowed	25	9.9	12	7.4	
**Education**
Primary school	107	42.3	36	22.2	0.001
Professional/secondary school	96	37.9	83	51.2	
University/high school	50	19.8	43	26.5	
Income*
Higher	29	11.5	32	19.8	0.001
Similar	48	19.0	37	22.8	
Lower	65	25.7	51	31.5	
Much lower	105	41.5	37	22.8	
Not willing to answer/unknown	6	2.4	5	3.1	
**Religiosity**
Secular	24	9.5	22	13.6	0.5
Traditional	169	66.8	98	60.5	
Religious	45	17.8	32	19.8	
Orthodox	15	5.9	10	6.2	
**Smoking**
Yes	127	50.2	78	48.2	0.7
No	126	49.8	84	51.9	
**Diabetes**
Yes	54	21.3	27	16.7	0.2
No	199	78.7	135	83.3	
**Dental insurance**
Yes	46	18.2	38	23.5	0.2
No	207	81.8	124	76.5	
**Dentist visit last year**
Yes	156	61.7	103	63.6	0.7
No	97	38.3	59	36.4	
Dentist	143	56.5	92	58.8	0.9
Dental hygienist	83	32.8	60	37.0	0.4
**Brushing teeth**
Yes	210	82.9	146	90.1	0.04
No	43	17.1	16	9.9	

*Compared to the national average income.

The results showed that among the irradiated group, 58 subjects (23%, 95% CI 18–28%) demonstrated complete edentulousness (six missing sextants) or insufficient teeth for CPI scoring as compared to 21 subjects (13%, 95% CI 8–19%) among the non-irradiated group (*p* = 0.01). Hence, CPI scores were recorded among 336 subjects (195 irradiated and 141 non-irradiated). Comparison of number of missing sextants yielded a median of 2 missing sextants among the 253 irradiated subjects, as compared to a median of 1 sextant among the 162 non-irradiated subjects (*p* < 0.001) (not shown).

As presented in Table 2, 162 subjects (48.2%) demonstrated “deep” periodontal pockets (operationally defined as periodontal disease). Strong and significant inverse relationships between periodontal disease and the two socioeconomic variables (education and income) were observed (*p* < 0.001). Smoking and diabetes were associated with increased periodontal disease (*p* = 0.05 and *p* = 0.02, respectively). Significantly more irradiated subjects had periodontal disease as compared to the non-irradiated subjects (54.4 vs. 39.7%, respectively; *p* = 0.008).

**Table 2:** **Percentage and odds ratio of “deep” periodontal pockets among study population by selected variables**.

	Total	Individuals with “deep” periodontal pocket		OR	95% CI	*p*
		*n*	%	
**Total**	336	162	48.2			
**Gender**
Male	149	69	46.3	1.0		
Female	187	93	49.7	1.15	0.74–1.76	0.5
**Age**
49–54	62	29	46.8	1.0		
55–59	147	64	43.5	0.88	0.48–1.59	0.7
60–64	102	54	52.9	1.28	0.68–2.41	0.4
65–72	25	15	60.0	1.71	0.66–4.38	0.3
**Family status**
Married	257	122	47.5	1.0		
Single/separate/divorced	52	25	48.0	1.02	0.56–1.86	0.9
Widowed	27	15	55.6	1.38	0.62–3.13	0.4
**Education**
Primary school	98	63	64.3	1.0		
Professional/secondary school	154	77	50.0	0.56	0.33–0.93	0.03
University/high school	84	22	26.2	0.20	0.10–0.37	<0.001
Income*
Higher	56	16	28.6	1.0		
Similar	73	30	41.1	1.74	0.84–3.73	0.14
Lower	90	46	51.1	2.61	1.30–5.43	0.008
Much lower	107	66	61.7	4.02	2.03–8.27	<0.001
**Religiosity**
Secular	42	15	35.7	1.0		
Traditional	208	105	50.5	1.84	0.93–3.72	0.08
Religious	65	33	50.8	1.86	0.84–4.18	0.13
Orthodox	21	9	42.9	1.35	0.46–3.95	0.6
**Smoking**
No	180	78	43.3	1.0		
Yes	156	84	53.9	1.53	0.99–2.35	0.05
**Diabetes**
No	274	124	45.3	1.0		
Yes	62	38	61.3	1.92	1.10–3.40	0.02
**Dental insurance**
No	261	137	52.5	1.0		
Yes	75	25	33.3	0.45	0.26–0.78	0.004
**Dental visit last year**
No	110	56	50.9	1.0		
Yes	226	106	46.9	0.85	0.54–1.34	0.5
**Brushing teeth**
No	15	7	46.7	1.0		
Yes	321	155	48.3	1.07	0.38–3.01	0.9
**Irradiation**
No	141	56	39.7	1.0		
Yes	195	106	54.4	1.81	1.17–2.81	0.008

*Compared to the national average income.

Periodontal health status according to CPI and irradiation status yielded a higher mean number of sextants with deep periodontal pockets (1.31 sextants of the “average mouth”) among the irradiated group compared to the control group (0.98 sextants) (data not shown).

Table 3 shows three models describing the association between radiation and periodontal disease. For ever being exposed to irradiation (part A of Table 3), the Odds Ratio (ORs) ranged from 1.8 (95% CI 1.17–2.81) to 1.6 (95% CI 1.01–2.57) when adding education and smoking to the model.

**Table 3:** **Factors associated with “deep” periodontal pockets – multivariate logistic regression results; (A) for irradiation yes versus no and (B) by dose**.

	Model I		Model II		Model III^a^	
	OR	95% CI	OR	95% CI	OR	95% CI
**A**						
**Irradiation**
Yes vs. No	1.81	1.17–2.81	1.62	1.0–2.57	1.61	1.01–2.57
**Education**						
Professional/secondary school vs. primary school			0.62	0.36–1.04	0.58	0.33–0.98
University/high level school vs. primary school			0.21	0.11–0.40	0.20	0.10–0.39
**Smoking**
Yes vs. No					1.65	1.04–2.61
**C statistic and 95% CI**	0.571 [0.519–0.624]		0.665 [0.612–0.725]		0.679 [0.623–0.735]	
**B**						
**Dose to the salivary gland**
(increase of 1 Gy)	2.18	1.27–3.81	1.97	1.11–3.50	1.95	1.10–3.50
**Education**
Professional/secondary school vs. primary school			0.62	0.36–1.05	0.57	0.33–0.98
University/high level school vs. primary school			0.22	0.12–0.42	0.21	0.11–0.40
**Smoking**
Yes vs. No					1.66	1.05–2.63
**C statistic and 95% CI**	0.594 [0.538–0.650]		0.670 [0.616–0.730]		0.688 [0.632–0.745]	

*^a^History of diabetes and dental insurance were initially included in the models. They were removed using backward elimination procedure since there were not significantly associated with periodontal disease in both models. *p* Values for Hosmer and Lemeshow Goodness of Fit test were 0.83 and 0.82 for A and B, respectively*.

A second analysis that included quantification of the risk by dose absorbed in the salivary gland (Part B of Table 3), showed an OR of 1.95 per 1 Gy (95% CI 1.10–3.50) controlling for education and smoking. Compared to the linear dose model, the linear quadratic dose-response model did not significantly improve the fit for the model including the dose to the salivary gland (*p* = 0.9). Education and smoking were also found to be independently and significantly associated with periodontal disease. A protective effect of 80% for having periodontal disease was found among those with higher education (95% CI 0.1–0.39). The two socioeconomic variables, education and income, were highly correlated and therefore, only one of them was inserted to the model. Smoking increased the risk for periodontal disease by 65% (95% CI 1.04–2.61). Non-significant increased and decreased odds ratios were found for diabetes and dental insurance (OR = 1.70 95% CI 0.94–3.10 and OR = 0.58 95% CI 0.33–1.03, respectively) (data not shown).

## Discussion

The present study suggests that childhood exposure to low–moderate doses of ionizing radiation (0.2–0.4 Gy) might be associated with later outcomes of dental health. Elevated risks among irradiated individuals were found for ever being exposed to radiation (irradiated vs. non-irradiated) as well as for an increment of 1 Gy absorbed by the salivary gland. Irradiated subjects had more missing sextants and higher levels of deep periodontal pockets as compared to the control group.

It should be noted that at an adult age several factors could lead to tooth loss, including dental caries, periodontal disease, orthodontic treatment, trauma, etc. Dental diseases are also recognized as being strongly related with socioeconomic variables (24–26). In the present study, lower level of education was found to be associated with increased risk for periodontal disease. Smoking has been established as one of the most significant risk factors in the development and progression of periodontal disease and was also found as an independent risk factor in the present study. Indeed, the American Academy of Periodontology includes tobacco cessation as a part of periodontal therapy (2, 27). Controlling for all these variables, past irradiation remained as an independent risk factor for higher level of periodontal disease.

Psycho-social factors, including trauma and immigration, are known to influence preventive behavior, such as oral hygiene and dental service attendance hence potentially affecting oral health (28). Insufficient or inadequate coping resources undermine the capacity for healthy behavior and thereby may exacerbate lifestyles known to potentiate periodontal disease. These include neglect of oral hygiene, changes in diet, and increase in smoking (29). In our analysis, we controlled for some of these components (education and smoking) and found that the impact of radiation remained an independent risk factor for periodontal health.

Several plausible biological explanations have also been offered for this association. A direct pathophysiological impact on host resistance, affecting the immune system, has been suggested. Salivary cortisol levels were found to be higher among people exhibiting severe periodontitis with high levels of stress (30). Close relationships between the extent and severity of periodontitis and salivary levels of stress-related hormones, cortisol, and dehydroepiandrosterone have been demonstrated (31).

Thus, it is important to consider life-course psycho-social, environmental, and economic in studies that assess oral health, specifically among immigrant societies (25, 32).

This is relevant to our study since the exposed (TC) group underwent psychological and socially distressing experience of having their hair painfully epilated by shaving and waxing before irradiation, followed by the stress and social stigma of alopecia (16). However, both study groups originated from the same cultural and socioeconomic background and all had experienced the potentially traumatic consequences of immigration. Therefore, we believe that these groups are similar and comparable.

The level of deep periodontal pockets (above 6 mm) seen among 40% of the non-irradiated subjects in our study is higher than the level in the total adult population, as reported by Petersen and Ogawa (4), but similar to older populations in their study. As examples, for the USA, 32% older people with deep periodontal pockets were reported, for Germany 40%, Finland 27%, Estonia 69%, and Denmark 20%. Moreover, the same authors also note that poor periodontal disease status is linked to low income or to low education (4). The present study consists of an older and poorer population.

In addition, it adheres with recent data that the global burden of oral conditions, including severe periodontal disease, has increased from 1990 to 2010 (5).

Among the present study population, the level of deep periodontal pockets was found to be higher among the irradiated subjects as compared to the non-irradiated subjects (54 vs. 40%). Prevalence of periodontal disease at older ages may induce dental and general health deterioration (4). Therefore, the findings of our study might have important implications for secondary (early detection of periodontal disease) and primary prevention (taking measures for preventing further deterioration) in irradiated populations.

Among the advantages of this study is the large irradiated cohort, the comparability of the exposed and the non-exposed groups with respect to demographic variables, the long follow-up period and the individual dental examinations, rather than relying on self-reported data or medical records. Another advantage of the TC studies is the availability of individual dosimetry for different organs. Schafer et al. (33) and Lubin et al. (34), who investigated the impact of uncertainties in the TC studies, concluded that the possible measurement error in dosimetry has a minimal effect on dose–response estimation and inference.

However, this study has some limitations primarily the incomplete compliance rate (79 and 48% between irradiated and non-irradiated individuals, respectively) that might lead to selection bias. Nevertheless, the non-significant differences observed between the participants and non-participants in demographic and other variables derived from the refusal questionnaire as well as the association of known risk factors with periodontal disease (e.g., education and smoking) that was found in this study support the validity of our results. It is also important to note that these compliance rates are acceptable nowadays in most epidemiological studies (35). The lack of information on additional possible confounders (e.g., exposure to radiation after childhood exposure, nutritional patterns, alcohol consumption, osteoporosis, and obesity) is another limitation of this study. High-dose radiation given as treatment for cancer is not a confounder in this study since individuals who developed a malignant disease were excluded from the study. The impact of diagnostic procedures that involve very low doses of radiation, if exists is probably minimal compared to the childhood exposure to moderate doses received for the treatment of TC. Regarding nutrition and alcohol consumption, both groups are composed of North African Jews who have very similar nutritional habits and only five participants reported on regular alcohol consumption. Therefore, it seems that the lack of adjustment for these factors could not have a significant influence on the results.

The conclusions of several studies, which have investigated the associations between dental radiology and adverse effects, are ambiguous (36–39). Recent research (40) has indicated that other low dose irradiations to this anatomical area, such as brain CT, may also be of adverse potential. Medical and dental radiology is a very common and prevalent diagnostic tool. The findings of our study suggest that childhood exposure to low–moderate doses of ionizing radiation might be associated with later outcomes of dental health. Properly and wisely administered radiographs, based on knowledge and experience, remain to be professionally necessitated and justified. Therefore, adherence to professional guidelines (41), recommending the appropriate use of dental radiographs, and consideration of multiple exposure, especially techniques which utilize relatively higher doses of radiation (e.g., CT), and cumulative effects over time, should be emphasized.

## Author Contributions

Substantial contributions to the conception or design of the work; or the acquisition, analysis, or interpretation of data for the work (SS, AC, HS-C, JM, TA, HE-N, YV). Drafting the work or revising it critically for important intellectual content (SS, AC, HS-C, YV). Final approval of the version to be published (SS, AC, HS-C, JM, TA, HE-N, YV). Agreement to be accountable for all aspects of the work in ensuring that questions related to the accuracy or integrity of any part of the work are appropriately investigated and resolved (SS, AC, YV).

## Conflict of Interest Statement

The authors declare that the research was conducted in the absence of any commercial or financial relationships that could be construed as a potential conflict of interest.
